# Patterns of Smoking and Snus Use in Sweden: Implications for Public Health

**DOI:** 10.3390/ijerph13111110

**Published:** 2016-11-09

**Authors:** Lars Ramström, Ron Borland, Tom Wikmans

**Affiliations:** 1 Institute for Tobacco Studies, Kanalvägen 17, SE-18338 Täby, Sweden; 2 Melbourne School of Population and Global Health, University of Melbourne, 235 Bouverie St, Carlton Victoria 3053, Australia; Ron.Borland@cancervic.org.au; 3 Nigel Gray Fellowship Group, Cancer Council Victoria, 615 St. Kilda Road, Melbourne, Victoria 3004, Australia; 4 LiQuSa-Research, Industrial Business Residence, Örsätter, SE-597 94 Åtvidaberg, Sweden; tom@liqusa.com

**Keywords:** public health, snus, smokeless tobacco, smoking cessation, tobacco control, harm reduction

## Abstract

There has been concern that the availability of alternative less harmful forms of nicotine might inhibit smoking cessation and/or encourage those who would not otherwise have smoked to do so. The plausibility of such effects can be best assessed by looking at population trends in use of smoking in relation to alternatives. This paper looks at the relationships between snus use and smoking in Sweden. Analyses are based on a data set for the period January 2003 to February 2011 from a long-term study covering nationally representative samples of the Swedish population aged 18–79, with a total study population of 60,675 individuals. Questionnaires made it possible to identify detailed tobacco use categories and use trajectories. The results showed that uptake of snus use is much more common in males than females. Those who began daily tobacco use using snus were much less likely to subsequently take up smoking than those who had not, both among males (17.6% vs. 45.9%), and females (8.2% vs. 40.2%). Further, among those who started using snus after starting as smokers, 76.3% of men and 71.6% of women had stopped smoking completely, including 31.5% of the men and 28.6% of the women who had quit all forms of tobacco. Indeed, those who were primary snus users were also more likely to have quit altogether than those who only ever smoked. Snus was also reported as the most common smoking cessation aid among men and yielded higher success rates than nicotine replacement therapy and other alternatives. As conclusions, snus has both contributed to decreasing initiation of smoking and, when used subsequent to smoking, appears to facilitate smoking cessation. All these effects suggest that the availability and use of snus has been a major factor behind Sweden’s record-low prevalence of smoking and the lowest level of tobacco-related mortality among men in Europe.

## 1. Introduction

The ultimate goal for tobacco control is generally stated as “A tobacco-free world.” However, there is no reasonable prospect of achieving this in the foreseeable future. As a result, many experts believe that there needs to be a focus on minimizing tobacco-related morbidity and mortality among present tobacco users [1,2]. This is particularly important for disadvantaged people, who evidence shows are less likely to be able to quit smoking successfully and are more likely to take it up [3].

For many years, there has been a debate about the threats and opportunities associated with lower harm forms of nicotine delivery, such as snus (the Swedish kind of low-toxicant smokeless tobacco). In recent years, the rise of vaporized nicotine products (electronic cigarettes) has sparked a new debate in this field. It is still too early to know exactly how this will turn out, but some insights into likely effects can be gained from the emergence of snus in Sweden since the 1970s. New manufacturing methods for snus were developed by the then government-owned tobacco monopoly to minimize levels of toxicants. The epidemiology is now clear: snus is markedly less harmful than smoking; indeed, there are no clearly established causes of premature death associated with its use [2,4,5,6], except for a potentially increased risk of fatality after acute myocardial infarction [7,8]. Indeed, neither oral or pancreatic cancer which have been linked to other smokeless tobacco sources are associated with snus [9,10]. Snus does not contain the combustion products in cigarettes and other smoked tobacco products. By contrast, dirtier forms of smokeless tobacco do result in clear adverse health effects [11]. These finding have helped to establish that nicotine itself is a minor cause of the morbidity and mortality related to tobacco use [12]. Therefore, nicotine-delivery products can be used without most of the serious health effects of smoking if the nicotine is not accompanied by high levels of disease-producing toxicants as it is in smoking. This principle is already an established practice in the use of nicotine replacement therapy (NRT)-products as a short-term means of quitting smoking, but the snus story shows that this extends to prolonged use.

There is now a broad consensus that use of non-combustible low-toxicity oral tobacco products like Swedish snus instead of combustible tobacco, will yield beneficial effects for individuals who use snus instead of cigarettes [13,14]. For example, it has been estimated that users of products like Swedish snus have at least 90%–95% less tobacco-related mortality than cigarette smokers and that their reduction of life expectancy is consequently small [5,15]. Indeed, among those who quit smoking for snus, the health benefits are similar to that for people who quit smoking completely.

Notwithstanding, there are concerns that, on the population level, snus use might have negative health effects, such as increasing total prevalence of tobacco use, serving as a gateway to smoking, hampering smoking cessation and leading to dual use of cigarettes and snus [16,17,18].

At a population level, the net effect of snus use on health is determined not only by the health risks for individual users but also by the way it influences national smoking patterns. The aim of this study is to ascertain the impacts of snus use on both uptake and cessation of smoking as key elements determining the net public health impacts of snus use.

What do we know about the impact of snus on smoking? Sweden and Norway are the two countries with the longest history of snus use, and where most of the research comes from. In Sweden, precursors to the modern snus have a long history but were in decline, only used by old men by the 1970s. However, as a reaction to the scientific evidence about health risks of smoking emerging in the 1960s, Sweden was the first country to institute permanent government funding of public health education on smoking. This triggered an interest, especially among men, in switching to snus that was at that time cheaper than cigarettes. In addition, the Swedish government, while making no health claims, allowed snus to be taxed at a lesser rate than cigarettes. However, in recent years, taxes on snus have been raised, so it is now at least as expensive as cigarettes. Snus use now exceeds smoking among men and has done so since at least 1996 [19]. Among women, daily snus use remained low but in recent years has increased from 1% in 1996 to 4% in 2015. Over the same period, rates of daily smoking have declined from 23% to 11% among women and from 19% to 10% among men. Recently, the growth of daily snus use among men has stalled: rates were 19% both in 1996 and 2015 [20]. This may be due to the increased taxes on snus. The current rate of daily smoking among men is far below any other country in the European Union. EU statistics indicate an overall prevalence (men + women) of 11% for Sweden and, for the rest of EU, from 19% (Finland) to 38% (Greece) with an EU average of 28% [21]. The Swedish pattern of increasing snus use and declining smoking has also occurred in Norway [22].

The above evidence constitutes compelling evidence that snus has not led to more smoking. However, there have been many other tobacco control initiatives in Sweden and progress may have been even more rapid without snus. The question remains: What other evidence is useful in addressing whether snus encourages smoking and/or discourages quitting? The idea that snus might encourage smoking is the so-called gateway effect whereby use of one form of nicotine sensitizes users so they are more likely to try an alternative form, especially one that is more psychoactive, like smoking. This theory can never be demonstrated at the individual level, because we cannot observe the counterfactual case of those who would have smoked without snus but just happened to use it first from those who would not have smoked except for use of snus. However, at a population level, it is possible to disconfirm various forms of the gateway hypothesis. Indeed, the rise of snus concurrent with a decline in smoking disproves the strong version of gateway that snus is the major factor driving changes in smoking prevalence. The case against any gateway would be strengthened if the rate of decline in smoking was greater in populations where snus use was increasing compared to where it was not (as much) or was declining.

The gateway hypothesis predicts that transitions to smoking will be greater among those who use snus first as compared with those who do not. A finding of lower uptake among snus starters would be fatal to it. However, the prediction of greater uptake is not unique to the gateway hypothesis. A Norwegian study showed that overall primary snus users have social and demographic characteristics that predispose them to smoking [23], thus at least some of any over-occurrence of subsequent smoking among primary snus users could be due to this co-occurrence of risk factors (assuming these findings also apply in Sweden, which seems likely). Thus, both gateway and the co-determinants hypotheses predict greater uptake of smoking among primary snus users than among the rest of the population.

The other concern about snus and other alternative forms of nicotine is that they may be used to sustain smoking by hampering rather than facilitating smoking cessation. Here the focus is on secondary snus use. There would be a clear public health benefit if snus use facilitated smoking cessation, and harm if it inhibited it. On the individual level, it is not possible without randomized trials to conclusively demonstrate that snus facilitates smoking cessation, but at the population level, the randomized trials have to be replaced by population studies. This is particularly the case, if the way snus is used is more to replace smoking, at least temporarily, rather than being used as a form of short-term therapy under external control [24]. To establish effectiveness at a population level, there is a need for evidence both about cessation success in groups using an aid and the extent of usage. Further, it is possible to demonstrate whether snus use hampers quitting at the population level or not. If the rate of smoking cessation among those who have used snus is greater than the proportion quitting who have not, then the simple version of the hampering hypothesis is disproven. Again, finding lower overall quit rates in populations with high snus use would be consistent with hampering, but higher rates would not. Also, similar to uptake, a co-occurrence of determinants of use of snus and smoking could explain lower quit rates, but not higher ones.

This analysis leads to the following main research questions:

1. Is primary uptake of snus associated with increased or decreased uptake of smoking as compared to those who have never been regular snus users?
2. How does smokers’ uptake of snus use influence continuation or cessation of tobacco use? In particular, is snus use, particularly secondary use (to smoking), associated with increased or reduced success in smoking cessation?
3. Have the relationships between the two products changed over time?
4. How commonly is snus used as a cessation aid compared to other options, and how does it compare in terms of success rates?

## 2. Methods

### 2.1. Data Source

The study is based on a data set for the period January 2003 to February 2011 retrieved from *Your Country and Your Life (YCYL)*, a long-term study of the Swedish people's attitudes, values and behaviors in a large number of societal, political and personal areas such as different living conditions including tobacco use. The study was conducted by the research institute FSI (Study Group for Societal and Information Studies) and was continuously ongoing from 1971.

### 2.2. Sample

Each year, probability samples of the Swedish population aged 18−79 years were drawn from the Swedish Population Registry (*n* ≈ 13,000). Representativity of the samples was checked with regard to gender and age. Since 1993, each sample was split into sub-samples (*n* ≈ 300) for continuous data collection by weekly distribution of postal questionnaires except during summer and Christmas holidays.

### 2.3. Survey Procedures

There was a total of four postal reminders where the second contained a new questionnaire and the fourth an abbreviated questionnaire. Every fourth week there was a random telephone follow-up of respondents who had not filled in the questionnaire after the fourth reminder.

The average response rate after non-response analysis was around 65%. The data was weighted to the population estimates by gender, age, education and place of residence.

The retrieved data set contained 62,213 respondents. There were 16 respondents excluded because they lacked registered value of either sex or age. Another 1522 were excluded because they did not have complete answers on tobacco issues. The current study population contains 60,675 respondents. Data analyses were performed using SPSS 13.0 for Windows (Chicago, IL, USA).

### 2.4. Measures

The data set from the YCYL study contained both demographic and tobacco-related items.

The demographic items included gender, date of birth, education, employment, place of residence, living conditions, national origin, income and financial assets. Register data for age, gender and place of residence were included and used to check the validity of the self-reported data.

The tobacco-related items included five basic questions: Q1. Do you now smoke: Daily, Occasionally, Not at all? Q2. Have you previously smoked: Daily, Occasionally, Never at all? Q3. Same as Q1 but for snus use. Q4. Same as Q2 but for snus use. Q5. Which onset came first: Smoking or snus use? (asked of those answering “Daily” to Q1 and/or Q2 in combination with “Daily” to Q3 and/or Q4). Other items covered time to first cigarette/snus after waking up, quantity of cigarettes/snus per day, age of initiation to daily smoking/snus use, desire to quit smoking, number of smoking cessation attempts, and cessation aid used at most recent attempt.

### 2.5. Data Processing

Detailed categories of tobacco use at the time of the survey were established based on the answers to the questions regarding smoking frequency.

Individual initiation profiles and tobacco use trajectories were established such that tobacco use can start either with smoking or snus use and that the subsequent progression of tobacco use can include sustained use, initiating the other product and quitting one or both. Based on reported daily use of each and which was used first for daily users of both, the following five categories were established: “Primary daily smoking, no daily snus use,” “Primary daily smoking, secondary daily snus use,” “Primary daily snus use, no daily smoking,” “Primary daily snus use, secondary daily smoking,” “No daily tobacco use.” NB: Defining initiation with respect to onset of occasional use would result in categories containing a heterogeneous mixture of subgroups that are too disparate to make sense of the data. By cross-tabulation of initiation profile with tobacco use at the time of the survey, it is possible to identify multi-step trajectories that individual tobacco users have followed from initiation to the time of the survey.

Since the prevalence of tobacco use is determined both by cessation and initiation practices, we addressed the question of changes over time in primary initiation. Data regarding decade of birth were used to establish cohort-specific profiles of primary initiation for people born in five consecutive decades. Inter-cohort comparisons will then give an insight into the changes over time in the primary initiation practices.

At various points in the manuscript, we report percentages of smokers as a function of whatever reference group (denominator) we are interested in. In some cases, where the denominator is “ever smokers,” the results are reported as quit ratios; i.e., the percentage of ex-smokers as a function of ever smokers.

### 2.6. Ethical Considerations

Full review by a research ethics committee has not been necessary for this type of data collection according to the rules of the Swedish Law on Research Ethics Review [25].

## 3. Results

### 3.1. Primary Initiation of Tobacco Use According to Changes over Time in the Population

**Figure 1:**
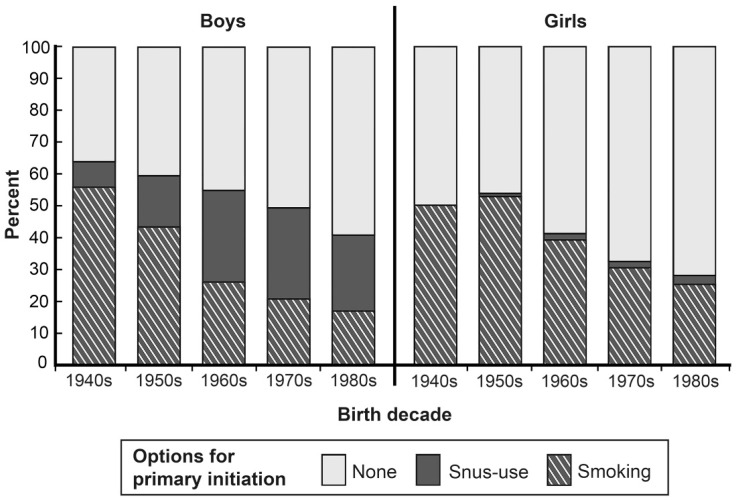
Changes over time concerning primary initiation. The bars represent cohorts from five consecutive birth decades. The segments show the proportions of different options for primary initiation in each cohort.

In order to analyze patterns of primary initiation at different periods in the past, we have studied separate birth cohorts with respect to the proportions between the three options: Primary initiation of daily smoking, Primary initiation of daily snus use, and No initiation of daily tobacco use. The sequence of cohort-specific data shown in Figure 1 demonstrates how initiation practices in the population have changed over time.

Among males born in the 1940s, a large proportion initiated daily tobacco use through smoking and a rather small proportion through snus use. Among those from later birth decades, primary initiation of daily snus use steadily increased along with decreased primary initiation of daily smoking and increased proportions never becoming daily tobacco users. In the cohort born in the 1980s, a majority of the boys never became daily tobacco users, and most of the boys who did initiate daily tobacco use started with snus use, with very few starting with smoking.

Among those born in the 1940s, primary initiation of smoking was more common among boys than girls, and primary initiation of snus use in girls was virtually non-existent. Among those born in the 1950s and later, primary initiation of smoking was more common in girls than boys, and the decrease from cohort to cohort was less pronounced in girls than in boys. Girls born in the 1950s were the first ones to show an appreciable level of primary initiation of snus use and this was the cohort in which overall tobacco use peaked. There have been some increases in primary initiation of snus in younger cohorts, but rates are still rather low (Figure 1).

### 3.2. Initiation of Daily Smoking According to Primary Daily Snus Use

Table 1 presents aggregated data from YCYL 2003−2011 in a way that makes it possible to compare differences in initiation of daily smoking as a function of prior daily snus use.

Overall, 40.8% (95% confidence interval (CI) 40.2–41.4) of the men (11,557 of 28,302) initiated daily smoking. There were 5021 (4144 + 877) primary daily snus users, and 44.5% (12,601) never daily tobacco users. This means there were 23,281 (28,302–5021) non-primary snus users; i.e., primary smokers and never tobacco users. Among the 5021 primary snus users there were 877 men, i.e., 17.6% (95% CI 16.4–18.6), who initiated daily smoking. Among the 23,281 non-primary snus users there were 10,680 (6943 + 3737) men, i.e., 45.9% (95% CI 45.3–46.5), who initiated daily smoking, a markedly higher figure.

Overall, 39.5% (95% CI 39.0–40.0) of the women (12,798 of 32,373) initiated daily smoking. Correspondingly, among the 527 (484 + 43) women with primary daily snus use, there were 43, i.e., 8.2% (95% CI 5.8–10.5), who initiated daily smoking. Among the 31,846 (32,373 − 527) women without primary daily snus use, there were 12,755 (11,794 + 961), i.e., 40.2% (95% CI 39.7–40.7), who initiated daily smoking—over four times the level in primary daily snus users.

Looked at another way, among the total of 11,557 men who ever initiated daily smoking there were just 877, i.e., 7.6% (95% CI 7.1–9.1) who proceeded to smoking from daily snus use (i.e., were primary snus users). The corresponding proportion among women was less than 0.1% (43 of 12,798).

Figure 1 shows that **primary** initiation of daily snus use has been increasing over time. Table 2 shows that the propensity for **secondary** initiation of daily snus use has also been increasing among men. This is occurring in a context of a massive decline in primary uptake of smoking and a small decline in secondary uptake. Members of later-born cohorts have lower age at the time of the survey and have therefore had a shorter time available for quitting smoking as reflected by the downward figures for quit ratios. However, there is an upward trend in the difference in quit ratios between primary smokers with and without uptake of secondary snus use. That is, use of snus in established smokers is associated with increased quitting in the latter cohorts wherein snus use is more prevalent. This observation lends further support to the hypothesis that snus use facilitates cessation, as opposed to hamper it. The first column of the table shows that the move away from primary uptake of smoking occurred in the cohort born in the 1960s; i.e., those who started to use tobacco from the mid-1970s to the end of the 1980s. It is notable that even in the context of this massive shift in initial use patterns, in all cohorts the proportion of primary smoking uptake was always greater among non-primary snus users than among primary ones.

### 3.3. Quitting Smoking According to Uptake of Daily Snus Use

Table 1 also presents data regarding quitting smoking and tobacco use at the time of the survey. In the total population (all initiation profiles), the proportion of daily smokers among males at the time of the survey (rows a + b + c) was 12.3% (95% CI 11.9–12.7) and the proportion of daily snus users (rows a + d + g) was 20.2% (95% CI 19.7–20.7). Corresponding values for females were 14.9% (95% CI 14.5–15.3) and 2.8% (95% CI 2.6–3.0). These data represent means over the data collection period 2003–2011.

Among the 10,680 male primary daily smokers, 34.9% (95% CI 34.0–35.8) had later taken up secondary daily snus use. Among women, the corresponding proportion was 7.5% (95% CI 7.0–8.0). Those who had taken up secondary snus use constituted 42.7% (95% CI 41.7–43.7) of all “ever daily snus users” in men and 64.6% (95% CI 62.2–67.0) in women. Table 1 also shows the proportion of ever daily smokers who had quit smoking in each of those different subgroups.

Among men with secondary daily snus use, 86.9% (95% CI 85.8–88.0) had ceased smoking daily (rows d–i) either quitting smoking completely (rows g–i) or reducing to occasional smoking (rows d–f). The corresponding proportion among women was 86.4% (95% CI 84.2–88.6). The proportion who had quit smoking completely was 76.3% (95% CI 74.9–77.7) among men and 71.6% (95% CI 68.7–74.5) among women. The corresponding proportions of secondary snus users who had quit all tobacco use (snus and smoking) were 31.5% (95% CI 30.0–33.0) among men and a similar 28.6% (95% CI 25.7–31.5) among women.

Among men without secondary daily snus use, 59.9% (95% CI 58.7–61.1) had ceased smoking daily. The corresponding proportion among women was 60.2% (95% CI 59.3–61.1). The proportion who had quit smoking completely was 55.3% (95% CI 54.1–56.5) among men and 53.6% (95% CI 52.7–54.5) among women.

Overall, secondary snus use is associated with success in smoking cessation. As shown in Table 2, this is true in all birth cohorts, especially with the larger proportion of secondary snus users.

Levels of cessation of smoking among primary snus users with uptake of smoking were intermediate (76.1% for men and 60.4% for women). Turning now to dual use, among men with secondary daily snus use, 18.9% (95% CI 17.6–20.2) had some kind of current use of both smoking and snus use (rows a + b + d + e). The corresponding proportion among women (rows j + k + m + n) was 21.7% (95% CI 19.1–24.3). More than half of these dual users had reduced from daily to occasional use of one or both products. In the total population, the proportion with daily use of both products was 1.68% (95% CI 1.53–1.83) among men and 0.23% (95% CI 0.18–0.21) among women.

Table 3 presents an overview of smoking cessation among daily smokers with different experience of daily snus use, this time reported in terms of quit ratios rather than percentages. Quit ratios are shown separately for “never” vs. daily snus users.

### 3.4. Self-Treatment Smoking Cessation: Different Cessation Aids and Their Effectiveness

**Figure 2:**
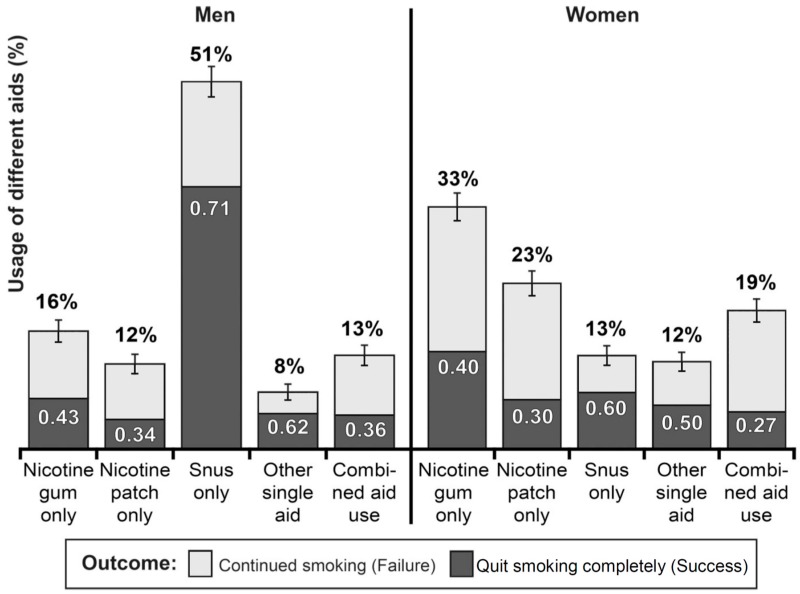
Self-treatment smoking cessation/quit attempts with use of different self-administered cessation aids and outcome of these quit attempts. The height of each bar illustrates the percentage of quit attempts that were made with the aid(s) in question, indicated numerically at the top of each bar by percentages adding up to 100% for each set. The segments of each bar represent outcome with the aid in question—failure (light-grey) or success (dark-grey). The numerical data in the dark-grey segments indicate the proportion of successful quit attempts for each cessation aid.

Both among men and women, 93% of all ever daily smokers reported that they had made at least one attempt to quit smoking. Most of them indicated that they had not had any professional assistance. However, around 40% of these unassisted quit attempters, both men and women, reported self-treatment by use of some self-administered nicotine-delivery product as a cessation aid. These quit attempters have been grouped in categories according to the choice of aid at the most recent quit attempt. Figure 2 shows both the relative size of these aid-usage categories and the outcome of the quit attempts in each category.

Among men, snus is the most commonly used self-treatment product, but among women, nicotine gum or patch is more common. The outcome of quit attempts with different aids shows that use of snus as cessation aid yields higher success rates than any of the other alternatives—for both men and women.

The combination of high usage and high successfulness means that, among men, snus has been the most effective product for self-treatment smoking cessation. In Figure 2, the size of each dark-grey segment corresponds to the quantity of successful quit attempts in the category in question. Using the numerical values for usage and success rate in each category, it can be calculated that among men “Snus only” was used in 64% of the successful quit attempts, while nicotine gum was used in 12% and the patch in 7%.

## 4. Discussion

The findings of this study are overwhelmingly supportive of snus playing both a role in protecting against the uptake of daily smoking and of facilitating smoking cessation, thus becoming an important contributor to improved public health. Both in men and women, quit ratios are significantly higher among smokers with than without experience of daily snus use, and this was most pronounced in secondary snus users. In each subgroup of snus use, there is no significant difference in quit ratio between men and women, while the much lower prevalence of snus use in women produces a gender difference among all ever daily smokers (Table 3).

### 4.1. Role of Snus in Initiation of Tobacco Use

The data presented in Figure 1 show that during the last 50 years in Sweden there has been a gradual increase of primary initiation of snus use accompanied by decreasing initiation of primary smoking and increasing proportions without initiation of any kind of daily tobacco use. There have been concerns that an increase of snus initiation would lead to increasing total initiation of tobacco use. However, the data presented in Figure 1 show that this has not happened. Indeed, the proportion of primary daily snus users who have progressed to any daily smoking has also declined marginally over this period (Table 2).

The “gateway” hypothesis is more definitively refuted by the findings reported in Section 3.2 that, both among boys and girls, the proportion of those that ever started daily smoking was significantly lower among primary snus users (17.6% among boys, 8.2% among girls) than among those without previous snus use (45.9% among boys, 40.2% among girls). Furthermore, this effect occurs when analyzing by cohort, demonstrating it is not a factor of the declining tobacco market. These findings are remarkable, and they suggest even stronger preventive effects of snus than we found as the risk factors for primary uptake of snus and smoking likely overlap. These findings based on retrospectively established individual trajectories are also consistent with findings from other studies [26,27,28,29,30,31,32]. The EU Scientific Committee on Emerging and Newly Identified Health Risks [14] summarized the evidence by concluding that “*The Swedish data, with its prospective and long-term follow-up do not lend much support to the theory that smokeless tobacco (i.e., Swedish snus) is a gateway to future smoking.”* We would go further and conclude that the above findings and other factors completely discount gateway as a credible hypothesis, at least for snus use in Sweden. If any gateway exists, it is dwarfed by other factors which are net protective. The evidence strongly points towards snus playing a positive preventative role. We can think of no alternative explanation for the patterns observed here and in the bulk of the existing literature.

### 4.2. Role of Snus Use in Cessation of Smoking

Secondary initiation of daily snus use occurred in around one-third of male primary smokers and represented almost half of all snus initiation. Among women, the secondary snus initiators were fewer than among men, but they constituted a majority of all female snus initiators.

Both male and female secondary snus users had substantially higher rates of quitting smoking than smokers without a history of snus use (Table 1). This was true both for quitting daily smoking by reducing to occasional and for quitting smoking completely. These findings demonstrate that smokers’ uptake of snus use favors smoking cessation; at least some of this uptake is motivated by an interest in quitting smoking.

Almost one-third of secondary snus users eventually quit all snus use and became entirely tobacco-free. This finding refutes the common assumption that uptake of snus use would entail lifelong perpetuation of nicotine dependence, based on the belief that snus use is as addictive as smoking. However, the high quit rates from snus are consistent with both clinical and pharmacokinetic studies indicating that snus has a lower dependence potential than cigarettes [33].

Further, the likelihood of eventual cessation of smoking was also greater among primary snus users with secondary smoking than only smokers. This may be in part due to the dual use as well as to snus being reengaged with assisting in quitting smoking.

Our findings refute the concerns that have been raised that dual use would hamper motivation to quit smoking, again consistent with other studies [34,35].

There has been a widespread concern that uptake of secondary snus use among daily smokers would usually result in permanent dual use and increase the risk of tobacco-related morbidity and mortality above the risk of single product use [17]. However, we found that more than eight out of ten secondary snus users had quit daily smoking and that almost one-third of them had become completely free of daily tobacco use. Consequently, there are strong reasons to assume that “dual use” is usually a transient rather than permanent state or an endpoint. It could then be seen as part of a multistep behavioral change where primary daily smokers use secondary uptake of snus as a stepping-stone towards changing/quitting their tobacco use. At least some of those who were actual dual users at the time of the survey should therefore not be regarded as permanent dual users—many may well at that time have been in a transient state on their way to cessation. Unfortunately, we do not have data on duration of use or of ambitions to change to sole use of one product.

To get some sense of how the quit ratios found here compare with other countries, we have sourced data from a Eurobarometer report [21]. Sweden exhibits a higher overall quit ratio than any other EU country (0.76), followed by the Netherlands and Denmark (both 0.57) then Finland (0.56). The quit ratio for the EU as a whole is 0.44. NB: The Eurobarometer data are not directly comparable with those in our study, since they include both former occasional and former daily smokers in the calculation of quit ratios.

The importance of snus for smoking cessation is reinforced by the findings of outcomes of “self-treatment quit attempts,” i.e., made without professional assistance in terms of medical and/or psychological treatment/counselling (this constitutes most such attempts) [31]. Among men, snus was both the most used, while for women it was only a minor method. In both genders, snus was the cessation aid that yielded the highest success rates compared to NRT-products or any other option, and it did so by a substantial margin. We do not know whether this higher success rate is due to higher success on any individual attempt or to making more attempts using snus, but from a public health point of view, this is not critical. The net benefit of using snus as a cessation aid is clear.

The high proportion of quit attempters using snus combined with their high successfulness has resulted in a majority, almost two-thirds, of male aid users who have succeeded in quitting smoking by using snus. This is more than double that of nicotine gum and nicotine patch together. The high effectiveness of snus as smoking cessation aid, appears to be an important contributory factor behind the high quit ratios in Sweden, particularly among men, and it implies a potential for snus to facilitate real large-scale smoking cessation, provided that both the product itself and objective information about its properties is freely available [36]. Again, these findings are consistent with other studies [22,34] and the key conclusions in authoritative reviews [37,38].

### 4.3. Implications for Other Harm-Reduced Products

Finally, we comment on the plausibility of generalizing results from this study to other harm-reduced products, particularly vaping. We can see no good reasons why a similar scenario to that which we have found for snus use should not develop with vaping, as it also protects against smoking and facilitates cessation. Substitute use of either represents a minor sacrifice in enjoyment for a large reduction in risks to health, a sacrifice many have not been able or willing to make when the alternative is abstinence (a large loss of enjoyment). However, it is important to assess possible impacts of differences between the two products. On the one hand, vaping is behaviorally closer to smoking, and the extraordinary expansion of use, almost entirely among smokers, strongly suggests it is a more easily accepted alternative to smoking than snus in many countries, although perhaps not in Scandinavia or in South Asia and other places where smokeless tobacco use is widespread despite the non-harm-reducing nature of smokeless tobaccos in South Asia. Second, the health effects of snus use are now established [6], while we only have theoretical analysis to estimate the impact of vaping; therefore, less confidence can be used in promoting it as relatively safe. That said, snus use has developed without actively promoting its reduced harmfulness, so this may not make much difference. We do not know whether vaping will prove to be more or less addictive than snus use, so we cannot be sure about relative likelihood of abstinence versus long-term nicotine use. We also do not know what the impact will be of vaping non-nicotine aerosols, an area where there is no direct parallel. It is likely to increase the normalization of vaping, but whether it does this in a way that encourages greater use of nicotine or not is unclear.

On the down side, it is likely over time that there will be some increase in total nicotine use, as there has been for snus. If vaping turns out to be more attractive than snus use, as seems likely, total nicotine use may be somewhat greater. In our opinion, this is a small price to pay if vaping is at least as effective as a means of both preventing and facilitating transitions away from smoking.

## 5. Limitations

This study has focused on transitions related to daily use, rather than occasional use as has often been done in other studies. We believe this is a strength, as it is prolonged daily use that is the major health risk factor, and because it avoids relying on inferences whose foundations are uncertain because of the heterogeneity that is inherent in the term “occasional use.” Further, from a health standpoint, occasional use is only of real importance when it can be used as a predictor of subsequent daily use. We believe the focus on daily smoking is particularly important for understanding dual use. The term “dual use” has often been used to denote any combination of smoking and snus use, daily or non-daily, and this has sometimes resulted in categories that are too heterogeneous to allow distinct interpretation of the observed data. In the current study, we have therefore focused on one clearly defined and homogeneous category of dual use, namely daily use of both kinds. As our data shows, this is relatively rare, though we do not know how stable it is. However, given that most secondary snus use leads to cessation of smoking, at least some appears to be transitory. It would be instructive to gain a better understanding of trajectories of dual use.

We also acknowledge that the reports on which form of tobacco came first are based on self-report, in many cases decades after starting, and that there is likely some error. However, we can think of no systematic source of error, or any explanation of how error in memory could have influenced the extremely strong associations we have observed.

We also acknowledge that this study was restricted to one country. It does not show that gateway effects or inhibition of quitting cannot happen anywhere. However, if it were to occur, it strongly suggests that it would have social determinants and thus should be preventable through public policy means. That said, we would be surprised if the findings were not directly generalizable.

## 6. Conclusions

The findings of this study indicate that the widespread use of snus has had a major influence on the development of smoking habits in Sweden. It appears that snus has contributed to decreasing initiation of smoking rather than serving as a gateway to smoking. Smokers who have taken up snus use have quit smoking to a significantly greater extent than smokers without snus use, and a substantial proportion has eventually quit snus use as well and become tobacco-free. These effects have been consistent across five decades and very different frequencies of snus use. Prolonged daily dual use is uncommon and, in general, dual use appears to be a transient state that does not hamper motivation to quit smoking but serves as a stepping-stone to cessation. Both among men and women, quit ratios are significantly higher for those with than for those without a history of snus use. Snus is the most commonly used self-treatment aid for smoking cessation. Quit attempters using snus as a cessation aid have a significantly higher success rate than those using other aids. All these effects yield favorable consequences for public health, suggesting that snus has been a major factor behind Sweden’s record-low prevalence of smoking and its position as the country with Europe’s lowest level of tobacco-related mortality among men based on analysis of data from a WHO report [39,40].

## Figures and Tables

**Table 1:** Progression of tobacco use by profile of initiation of tobacco use.

Profile of Initiation							
Tobacco use		Primary daily smoking		Primary daily snus use		No daily	
at the time of the survey		no daily	secondary	no daily	secondary	tobacco	
		snus use	daily snus use	smoking	daily smoking	use	Total
*Men*		*n* = 6943	*n* = 3737	*n* = 4144	*n* = 877	*n* = 12,601	*n* = 28,302
Daily smoking (combined with)							
daily snus use	*(a)*	–	9.0% *n* = 337	–	15.9% *n* = 139	–	1.7% *n* = 476
occasional snus use	*(b)*	3.9% *n* = 273	0.9% *n* = 33	–	2.0% *n* = 18	–	1.1% *n* = 324
no snus use	*(c)*	36.3% *n* = 2517	3.2% *n* = 120	–	6.0% *n* = 53	–	9.5% *n* = 2690
Occasional smoking (combined with)							
daily snus use	*(d)*	–	8.4% *n* = 315	10.7% *n* = 443	10.3% *n* = 90	–	3.0% *n* = 848
occasional snus use	*(e)*	0.5% *n* = 31	0.6% *n* = 22	0.7% *n* = 29	0.8% *n* = 7	0.7% *n* = 83	0.6% *n* = 172
no snus use	*(f)*	4.1% *n* = 283	1.6% *n* = 59	2.1% *n* = 85	2.5% *n* = 22	3.7% *n* = 468	3.2% *n* = 917
*Quit daily smoking, now occasional smoking (sum of d +e + f)*		4.6% *n* = 314	10.6% *n* = 396	N/A	13.6% *n* = 119	N/A	2.9% *n* = 829
No smoking (combined with)							
daily snus use	*(g)*	–	43.8% *n* = 1636	58.4% *n* = 2421	37.3% *n* = 327	–	15.5% *n* = 4384
occasional snus use	*(h)*	1.0% *n* = 71	1.0% *n* = 37	3.5% *n* = 145	0.9% *n* = 8	1.7% *n* = 216	1.7% *n* = 477
no snus use *(tobacco free)*	*(i)*	54.3% *n* = 3768	31.5% *n* = 1178	24.6% *n* = 1021	24.3% *n* = 213	93.9% *n* = 11,834	63.7% *n* = 18,014
*Quit daily smoking, now completely*		55.3% *n* = 3839	76.3% *n* = 2851	N/A	62.5% *n* = 548	N/A	25.6% *n* = 7238
*smoke free (sum of g + h + i)*							
Total (a − i)		100%	100%	100%	100%	100%	100%
*Women*		*n* = 11,794	*n* = 961	*n* = 484	*n* = 43	*n* = 19,091	*n* = 32,373
Daily smoking (combined with)							
daily snus use	*(j)*	–	7.3% *n* = 70	–	7.0% *n* = 3	–	0.2% *n* = 73
occasional snus use	*(k)*	1.6% *n* = 183	1.8% *n* = 17	–	11.6% *n* = 5	–	0.6% *n* = 205
no snus use	*(l)*	38.2% *n* = 4511	4.4% *n* = 42	–	20.9% *n* = 9	–	14.1% *n* = 4562
Occasional smoking (combined with)							
daily snus use	*(m)*	–	11.2% *n* = 108	11.8% *n*=57	11.6% *n* = 5	–	0.5% *n* = 170
occasional snus use	*(n)*	0.3% *n* = 38	1.4% *n* =1 4	1.4% *n*=7	2.3% *n* = 1	0.3% *n* = 58	0.4% *n* = 118
no snus use	*(o)*	6.3% *n* = 748	2.2% *n* = 22	2.3% *n* = 11	2.3% *n* = 1	4.5% *n* = 863	5.1% *n* = 1645
*Quit daily smoking, now occasional*		6.6% *n* = 786	14.8% *n* = 144	N/A	16.2% *n* = 7	N/A	2.9% *n* = 937
smoking (sum of m + n + o)							
No smoking (combined with)							
daily snus use	*(p)*	–	41.3% *n* = 397	55.8% *n* = 270	27.9% *n* = 12	–	2.1% *n* = 679
occasional snus use	*(q)*	0.4% *n*=44	1.7% *n* = 16	4.5% *n* = 22	0.0% *n* = 0	0.6% *n* = 118	0.6% *n* = 200
no snus use *(tobacco free)*	*(r)*	53.2% *n* = 6270	28.6% *n* = 275	24.2% *n* = 117	16.3% *n* = 7	*94.6% n =* 18,052	*76.4% n =* 24,721
*Quit daily smoking, now completely*		53.6% *n* = 6314	71.6% *n* = 688	N/A	44.2% *n* = 19	N/A	21.7% *n* = 7021
smokefree (sum of p + q + r)							
Total (j − r)		100%	100%	100%	100%	100%	100%

*Note:* N/A inserted in cells where percentages for *Tobacco use at the time of the survey* by *Initiation profile* are not applicable. Dash (–) inserted in cells where data can only assume the value 0 (zero).

**Table 2:** Occurrence and effects of secondary snus use in different birth cohorts of men in Sweden.

Born in	Proportion of uptake of daily nicotine use that is primary smoking	Proportion of non-primary snus users becoming smokers	Proportion of primary snus users becoming smokers	Proportion of primary smokers who take up secondary daily snus use	Quit ratios (quitting smoking completely)	
					Primary smokers without daily snus use	Primary smokers with daily snus use
1940s	87.3%	60.4%	19.6%	34%	0.60	0.83
1950s	73.4%	53.8%	22.8%	40%	0.48	0.77
1960s	46.8%	36.6%	18.6%	41%	0.40	0.72
1970s	42.5%	28.7%	13.5%	45%	0.31	0.66
1980s	40.1%	21.8%	14.3%	46%	0.19	0.47

**Table 3:** Quit ratios for smoking—by gender and history of daily snus use. (Proportion of “Ever daily smokers” in different subgroups who have quit smoking completely at the time of the survey.)

	Men		Women	
	QR	95% CI	QR	95% CI
Never daily snus users (Men: *n* = 6943, Women: *n* = 11,794)	0.55	(0.54–0.56)	0.54	(0.53–0.55)
Secondary snus users (Men: *n* = 3737, Women: *n* = 961)	0.76	(0.75–0.77)	0.72	(0.69–0.75)
Secondary smokers (Men: *n* = 877, Women: *n* = 43)	0.63	(0.60–0.66)	0.44	(0.29–0.59)
All of any daily snus users (Men: *n* = 4614, Women: *n* = 1004)	0.74	(0.73–0.75)	0.70	(0.67–0.73)
All ever daily smokers (Men: *n* = 11,557, Women: *n* = 12,798)	0.63	(0.62–0.64)	0.55	(0.54–0.56)

*Note:* QR = Quit ratio, CI = Confidence interval.
